# Prevalence of vulvovaginal candidiasis, bacterial vaginosis and trichomoniasis in pregnant women attending antenatal clinic in the middle belt of Ghana

**DOI:** 10.1186/s12884-019-2488-z

**Published:** 2019-09-23

**Authors:** Dennis Gyasi Konadu, Alex Owusu-Ofori, Zuwera Yidana, Farrid Boadu, Louisa Fatahiya Iddrisu, Dennis Adu-Gyasi, David Dosoo, Robert Lartey Awuley, Seth Owusu-Agyei, Kwaku Poku Asante

**Affiliations:** 1 Kintampo Health Research Centre, P. O. Box 200, Kintampo, Bono East Ghana; 2 Department of Clinical Microbiology, School of Medical Science, College of Health Science, Kwame Nkrumah University of Science and Technology, Kumasi, Ghana; 3 Microbiology Department, Komfo Anokye Teaching Hospital, Kumasi, Ghana; 4 University of Health and Allied Sciences, Ho, Ghana

## Abstract

**Background:**

Vaginal infections usually caused by *Candida sp,* organisms responsible for bacterial vaginosis and *Trichomonas vaginalis* are associated with considerable discomfort and adverse outcomes during pregnancy and child birth. The study determined the prevalence of vulvovaginal candidiasis (VVC), bacterial vaginosis (BV) and trichomoniasis (TV) in pregnant women attending antenatal clinic at the Kintampo Municipal Hospital.

**Methods:**

A study adopted a cross sectional design and recruited 589 pregnant women after seeking their informed consent from September, 2014 to March, 2015. Semi-structured questionnaire were administered to participants and vaginal swabs were collected. The samples were analysed using wet mount method and Gram stain (Nugent criteria) for vaginal infection. Univariate and multivariate analysis were used to investigate association of risk factors to vaginal infections.

**Results:**

The overall prevalence of at least one vaginal infection was 56.4%. The prevalence of vulvovaginal candidiasis, bacterial vaginosis and trichomoniasis were 36.5, 30.9 and 1.4% respectively. Women with more than four previous pregnancies (OR: 0.27, 95% CI: 0.13–0.58) and those in the third trimester of pregnancy (OR: 0.54, CI: 0.30–0.96) were associated with a lower risk of bacterial vaginosis. Douching and antibiotic use were neither associated with VVC or BV.

**Conclusion:**

The prevalence of vaginal infections was high among pregnant women in the Kintampo area. There is the need for interventions such as adequate investigations and early treatment of vaginal infections to reduce the disease burden to avoid associated complications.

## Background

Reproductive tract infection (ReTI) is an important public health problem worldwide especially in developing countries [1]. Globally, the World Health Organization (WHO) reports an estimated 357 million new cases of curable reproductive tract infection (ReTI) or sexually transmitted infections (STIs) (syphilis, gonorrhea, chlamydia infection, and trichomoniasis) which occur annually in adults. These infections can be symptomatic or asymptomatic [2]. Common symptoms reported are vaginal discharge, itching, irritation, unpleasant odor and discomfort. The main effect of ReTI on pregnancy is preterm delivery [3, 4]. However, pelvic inflammatory disease (PID), spontaneous abortion [5], low birth weight, infertility, premature rupture of membrane (PROM) and miscarriage [6] have also been linked with BV. This study focused on the three most common ReTI which include vulvovaginal candidiasis (VVC), bacterial vaginosis (BV), and trichomoniasis (TV) which have been association with poor pregnancy outcomes but typically unattended to [3, 5, 6].

VVC is caused by *Candida spp* commonly *C. albicans, C. glabrata* and *C. tropicalis* [7]. *Candida spp* is asymptomatic in about 20 to 50% of healthy women [8–10]. Vaginal colonization have been attributed to a number of factors, including pregnancy, prolonged use of broad spectrum antibiotics and poor personal hygiene [10]. BV results from alterations of the normal flora in the vagina mainly dominated by lactobacillus species to an overgrowth of anaerobic species [11–13]*.* BV is the common cause of abnormal vaginal discharge in women of child bearing age [14–16] with prevalence between 6 to 54% [17–19]. Women with disrupted vaginal flora such as BV are more likely to acquire other infections such as herpes simplex virus type-2 (HSV-2) [15, 20], *Trichomonas vaginalis* [21], *Neisseria gonorrhoeae* and human immune deficiency virus (HIV) [22]. TV is caused by *Trichomonas vaginalis* responsible for an estimated 180 million infections per year, making it the most prevalent non-viral sexually transmitted pathogen worldwide [23]. The parasite is usually found in the vagina, cervix and periurethral gland [24]. Approximately, 25% of women with TV infection are asymptomatic [25]. Symptomatic patients experience signs and symptoms such as vulvovaginal erythema, dysuria, pruritus, edema, frothy yellow-gray or green vaginal discharge and an elevated pH (> 6).

In Ghana, studies on prevalence of vaginal infection were been carried out in urban settings where majority of the population are educated [26–28]. Kintampo a rural urban community, located in the middle belt of Ghana with high illiteracy rate provides a good setting to carry out ReTI studies to compare with that of urban settings. This present study was conducted to determine the prevalence of vaginal infection and its associated risk factors.

## Methods

### Study site and study design

A descriptive cross-sectional survey was carried out in the antenatal care unit (ANC) of the Kintampo Municipal Hospital (KMH) between September 2014 and March 2015. The KMH is located in the geographical centre of Ghana. The hospital has an Antenatal Clinic which serves, on average about 30 pregnant women each working day. The KMH laboratory runs routine test for the pregnant women, but the bacteriology work for this study was carried out in the Kintampo Health Research Centre (KHRC) Clinical Laboratory.

### Study population, participant’s selection and recruitment

The surveys were carried out thrice a week and the first eight to ten pregnant women in any trimester gave consent to be part of the study during their antenatal visit were enrolled into the study each day. The number recruited per day was limited to allow for adequate analysis of samples collected. Women who had pregnancy related complications or refused consent or had previously participated in the study were excluded from the study.

### Study procedure

After consent and recruitment, information on socio-demographic variables, obstetric and sexual history, symptoms of vaginal infection and reproductive risk factors were obtained using a semi-structured pretested questionnaire. As part of the study procedure, a trained midwife examined the vulva and documented the presence of any abnormality including as genital warts, genital ulcer, lesions and abnormal vaginal discharge. Two swabs were taken under aseptic conditions by a trained midwife from the posterior vaginal fornix. The swabs were transported cold to Kintampo Health Research Centre’s (KHRC) Clinical Laboratory within 10 min after sample collection. Samples are analyzed immediately upon reaching the lab to avoid drying of the specimen collected.

### Laboratory test processes

Samples were processed at the Bacteriology section of the Clinical Laboratory of KHRC. The Laboratory has a good internal quality assurance system and also participates in an external quality assessment programme for microbiology with National Institute of Communicable Diseases in South Africa. A Gram stain smear was prepared from one swab (labeled sample 1) then examined microscopically by qualified laboratory personnel for BV diagnosis, using the Nugent’s scoring system (Nugent’s criteria) [29]. A participant was declared positive for BV if her Nugent score was between 7 to 10 but negative if less than 7. Ten percent of the smears were re-read by another reader as quality control which was in agreement with the first reader. The second swab (labeled sample 2) from the same participant was also used for wet mount preparation to identify yeast cells (Budding/Pseudohyphae/Hyphae), pus cell, clue cells and *Trichomonas vaginalis*. The wet mount procedure involves adding few drops of normal saline to the swab, shaking gently to mix sample with the saline. A drop was placed on a glass slide, cover slipped and observed with X100 and X400 magnification.

The laboratory personnel were blinded to the clinical findings of the participants, or any other details, except participants’ study numbers and specimen collection dates.

### Samples size estimation

A sample size of 606 was obtained based on a reported 6.4% prevalence of BV among pregnant women attending ANC in Burkina Faso [17]. A higher prevalence of 10% BV was assumed in a predominantly rural setting such as Kintampo with 90% power and 95% confidence level taking into account 5% refusals the sample size provided 93 and 94% power to estimate the prevalence of VVC and TV respectively. The power was calculated using a known prevalence of 34.2% (VVC) and 1.4% (TV) in women attending gyaenacological and antenatal clinic in Accra, Ghana [30]. An assumed prevalence of 41 and 3.5% for VVC and TV was respectively used in the calculations.

### Data collection, management and analysis

Data were double entered using MS-access software (Microsoft Corporation Copyright 2003) and checked for consistencies. Data was analysed using STATA Version 14 (Stata Corp, TX USA). All categorical variables were summarized as proportions whilst continuous variables were summarized as means or median based on the distribution of the variables. Associations between categorical variables were explained using the chi-square test *P* < 0.05 was considered significant. Univariate and multivariate logistic regression models were used to identify potential risk factors of VVC and BV but not TV due to the low numbers identified. We assessed the association between BV, VVC and both BV and VVC infection with reported symptoms using the chi-square test.

## Results

### Characteristics of study participants

A total of 606 pregnant women were contacted to participate in the study but 97.9% (593/606) gave consent. The remaining 13 participants refused to consent to participate in the study. Out of the 593 consented participants, 589 had complete data on their demographics and vaginal samples whiles 4 participants gave information on only their demographic but refused vaginal swab collection. Demographic and obstetric characteristics of the participants are summarized in Table 1. The mean age of the women was 27 years (*N* = 593, range 12 to 54 years). Married women, Christians and employment status of participants were 68.8% (408/593), 65.6% (389/593) and 62.6% (371/593) respectively. Slightly above half of the participants had completed primary or Junior High School 52.5% (311/593) and in the second trimester of pregnancy 53.3% (316/593).

**Table 1:** Characteristics of study participants

Characteristics	*N* = 593 *n* (%)
Age group	
< 19	77 (13.0)
20–34	423 (71.3)
> 35	92 (15.5)
Missing	1 (0.002)
Marital Status	
Single	96 (16.2)
Cohabiting	88 (14.8)
Married	408 (68.8)
Missing	1 (0.002)
Religion	
Christians	389 (65.6)
Muslim	188 (31.7)
Others	16 (2.7)
Occupation	
Employed	371 (62.6)
Unemployed	222 (37.4)
Education	
No education	211 (35.6)
Primary & JHS	311 (52.5)
> SHS	71 (12.0)
Trimester	
First	81 (13.7)
Second	316 (53.3)
Third	196 (33.1)
Gravidae	
Primigravidae	145 (24.5)
2–4 gravidae	317 (53.5)
5 gravidae	131 (22.1)
History of spontaneous abortion	
Yes	99 (16.7)
No	494 (83.3)
Contraceptive Use	
Yes	168 (28.3)
No	425 (71.7)
Douching	
Yes	219 (36.9)
No	374 (63.1)
Antibiotic use in the past 2 weeks	
Yes	26 (4.4)
No	567 (95.6)
Sex frequency	
< 2 per week	424 (71.4)
> 2 per week	170 (28.6)
New sexual partners in the past 3 months	
Yes	1 (0.002)
No	588 (99.8)
Presence of abnormal vaginal discharge	
Yes	166 (28.2)
No	423 (71.8)
Genital Warts	
Yes	5 (0.8)
No	584 (99.2)

### Prevalence of VVC, BV and TV

**Fig. 1:**
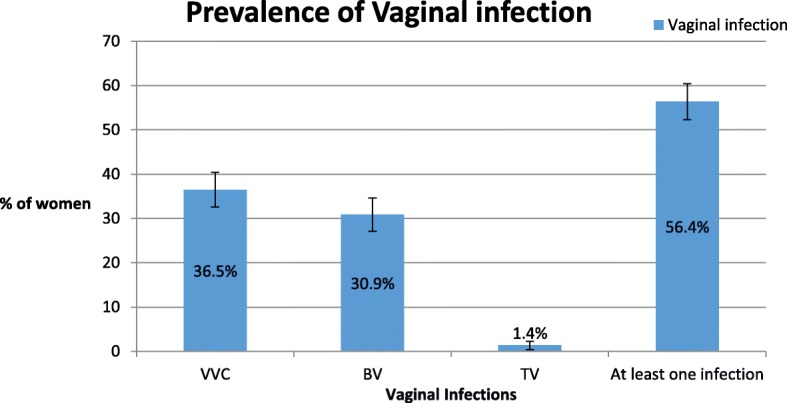
Prevalence of the vaginal infections among pregnant women (*N* = 589)

The prevalence of VVC, BV and TV among the pregnant women were 36.5% (215/589), 30.9% (182/589) and 1.4% (8/589) respectively. The prevalence of at least one of the three vaginal infections (VVC or BV or TV) was 56.4% (332/589) (Fig. 1).

### Risk factors for vulvovaginal candidiasis (VVC) and bacterial vaginosis (BV)

In VVC, univariate and multivariate were not significantly associated with all possible risk factors in the analysis (Table 2).

**Table 2:** Risk factors for vulvovaginal candidiasis (VVC)

	VVC		OR (Unadjusted)	*P*-value for OR	AOR (Adjusted)	*P*-value for AOR
	Yes *n* (%)	No *n* (%)				
Age group						
< 19	25 (32.1)	53 (67.9)	1.0		–	
20–34	158 (37.4)	264 (62.6)	1.27 (0.76–2.12)	0.365	–	–
35+	32 (36.0)	57 (64.0)	1.19 (0.63–2.26)	0.596	–	–
Marital Status						
Married	149 (36.9)	255 (63.1)	1.0		–	
Single	35 (36.1)	62 (63.9)	0.97 (0.61–1.53)	0.884	–	–
Cohabiting	31 (35.2)	57 (64.8)	0.93 (0.57–1.50)	0.770	–	–
Religion						
Christians	143 (36.9)	245 (63.1)	1.0		–	
Muslim	63 (34.1)	122 (65.9)	0.88 (0.63–1.28)	0.514	–	–
Others	9 (56.3)	7 (43.8)	2.20 (0.80–6.04)	0.125	–	–
Education						
No education	80 (37.9)	131 (62.1)	1.0		–	
Primary & JHS	112 (36.2)	197 (63.8)	0.93 (0.65–1.33)	0.699	–	–
> SHS	23 (33.3)	46 (66.7)	0.82 (0.46–1.45)	0.494	–	–
Occupation						
Unemployed	73 (32.7)	150 (67.3)	1.0		–	
Employed	142 (38.8)	224 (61.2)	0.77 (0.54–1.09)	0.139	–	–
Trimester						
First	30 (36.1)	53 (63.9)	1.0		–	
Second	110 (34.8)	206 (65.2)	0.94 (0.57–1.56)	0.821	–	–
Third	75 (39.5)	115 (60.5)	1.15 (0.67–1.96)	0.603	–	–
Gravidae						
Primigravidae	46 (31.9)	96 (68.1)	1.0		–	
2–4 gravidae	123 (39.2)	191 (60.8)	1.37 (0.90–2.08)	0.137	–	–
5 gravidae	46 (35.1)	85 (64.9)	1.15 (0.70–1.90)	0.578	–	–
History of spontaneous abortion						
No	176 (35.8)	315 (64.2)	1.0		–	
Yes	39 (39.8)	59 (60.2)	1.18 (0.76–1.85)	0.459	–	–
Contraceptive use before Pregnancy						
No	152 (36.0)	270 (64.0)	1.0		–	
Yes	63 (37.7)	104 (62.3)	1.07 (0.74–1.56)	0.698	–	–
Douche						
No	136 (36.7)	235 (63.3)	1.0		–	
Yes	79 (36.2)	139 (63.8)	0.9 (0.69–1.39)	0.919	–	–
Antibiotic use						
No	202 (35.9)	361 (64.1)	1.0		–	
Yes	13 (50.0)	13 (50.0)	1.79 (0.83–3.93)	0.149	–	–
Sex Frequency						
< 2 per week	161 (38.3)	259 (61.7)	1.0		–	
> 2 per week	54 (32.0)	115 (68.1)	0.76 (0.52–1.10)	0.146	–	–
Trichomoniasis						
No	211 (36.3)	370 (63.7)	1.0		–	
Yes	4 (50.0)	4 (50.0)	1.75 (0.43–7.08)	0.430	–	–

For BV, univariate analysis (Table 3) of marital status (single and cohabiting), primary & JHS education, occupation were independent risk factor for BV whiles age group (20–34, > 35), third trimester of pregnancy and multigravidae were associated with a lower risk. Douching, recent antibiotic use and sex frequency were not significant risk factors for BV. In multivariate analysis, only pregnant women in the third trimester of pregnancy and previous pregnancy of more than 4 were protective for bacterial vaginosis.

**Table 3:** Risk factors for BV

	BV Status		OR (Unadjusted)	*P*-value	AOR (Adjusted)	*P*-value for AOR
	Yes *n* (%)	No *n* (%)				
Age group						
< 19	36 (46.2)	42 (53.8)	1.0		1.0	
20–34	128 (30.3)	294 (69.7)	0.51 (0.31–0.83)	0.007	0.93 (0.51–1.68)	0.802
35+	18 (20.2)	71 (79.8)	0.30 (0.14–0.59)	< 0.001	1.02 (0.42–2.43)	0.969
Marital Status						
Married	103 (25.5)	301 (74.5)	1.0		1.0	
Single	45 (46.4)	52 (53.6)	2.53 (1.60–4.00)	< 0.001	1.60 (0.94–2.71)	0.083
Cohabiting	34 (38.6)	54 (61.4)	1.84 (1.13–2.99)	0.014	1.41 (0.84–2.35)	0.189
Religion						
Christians	127 (32.7)	261 (67.3)	1.0		–	
Muslim	48 (26.0)	137 (74.0)	0.72 (0.48–1.06)	0.100	–	–
Others	7 (43.7)	9 (56.3)	1.60 (0.58–4.39)	0.363	–	–
Education						
No education	52 (24.6)	159 (75.4)	1.0		1.0	
Primary & JHS	110 (35.6)	199 (64.4)	1.69 (1.14–2.50)	0.008	1.17 (0.76–1.79)	0.473
> SHS	20 (71.0)	49 (29.0)	1.24 (0.68–2.29)	0.474	0.80 (0.41–1.55)	0.503
Occupation						
Unemployed	84 (37.7)	139 (62.3)	1.0		1.0	
Employed	98 (26.8)	268 (73.2)	1.65 (1.16–2.36)	0.006	0.98 (0.64–1.50)	0.940
Trimester						
First	31 (37.3)	52 (62.7)	1.0		1.0	
Second	104 (32.9)	212 (67.1)	0.82 (0.50–1.36)	0.447	0.80 (0.47–1.34)	0.395
Third	47 (24.7)	143 (75.3)	0.55 (0.32–0.96)	0.035	0.54 (0.30–0.96)	0.035
Gravidae						
Primigravidae	64 (44.4)	80 (55.6)	1.0		1.0	
2–4 gravidae	99 (31.5)	215 (68.5)	0.58 (0.38–0.86)	0.008	0.67 (0.41–1.10)	0.114
5 gravidae	19 (14.5)	112 (85.5)	0.21 (0.12–0.38)	< 0.001	0.27 (0.13–0.58)	0.001
History of spontaneous abortion						
No	158 (32.2)	333 (67.8)	1.0		–	
Yes	24 (24.5)	74 (75.5)	0.68 (0.42–1.12)	0.134	–	–
Contraceptive use before present Pregnancy						
No	139 (32.9)	283 (67.1)	1.0		–	
Yes	43 (25.7)	124 (74.3)	0.71 (0.47–1.06)	0.090	–	–
Douche						
No	119 (32.1)	252 (67.9)	1.0		–	
Yes	63 (28.9)	155 (71.1)	0.86 (0.60–1.24)	0.421	–	–
Antibiotic use						
No	171 (30.4)	392 (69.6)	1.0		–	
Yes	11 (42.3)	15 (57.7)	1.68 (0.76–3.74)	0.202	–	–
Sex Frequency						
< 2 per week	130 (31.9)	290 (69.1)	1.0		–	
> 2 per week	52 (30.8)	117 (69.2)	0.99 (0.67–1.46)	0.965	–	–
Trichomoniasis						
No	179 (30.8)	402 (69.2)	1.0		–	
Yes	3 (37.5)	5 (62.5)	1.35 (0.32–5.70)	0.685	–	–
Candidiasis						
No	115 (30.7)	259 (69.3)	1.0		–	
Yes	67 (31.2)	148 (68.8)	1.02 (0.71–1.47)	0.917	–	–

### Association between vaginal infection and reported symptoms

Self-reported symptoms of vaginal infection were all significantly associated with vaginal infection using the chi-squared test (Table 4).

**Table 4:** Association between symptom and laboratory confirmed vaginal infection

Symptoms	Infection				*P*-value
	No infection (*n* = 259)	BV only (*n* = 115)	VVC only (*n* = 148)	BV and VVC (*n* = 67)	
Abdominal pain					
Present	52 (20.08)	30 (26.09)	28 (18.92)	27 (40.30)	0.002
Absent	207 (79.92)	85 (73.91)	120 (81.08)	40 (59.70)	
Pruritis					
Present	46 (17.76)	34 (29.57)	35 (23.65)	27 (40.30)	0.001
Absent	213 (82.24)	81 (70.43)	113 (76.35)	40 (59.70)	
Malodour					
Present	55 (21.24)	33 (28.70)	25 (16.89)	22 (32.84)	0.025
Absent	204 (78.76)	82 (71.30)	123 (83.11)	45 (67.16)	
Dysuria					
Present	22 (8.49)	11 (9.57)	11 (7.43)	14 (20.90)	0.013
Absent	237 (91.51)	104 (90.43)	137 (92.57)	53 (79.10)	
Altered discharge					
Present	84 (32.43)	49 (42.61)	43 (29.05)	38 (56.72)	< 0.001
Absent	175 (67.57)	66 (57.39)	105 (70.95)	29 (43.28)	
Some symptom (Abdominal pain, Pruritis, Malodour, Dysuria and/or Altered discharge)					
Yes	89 (34.36)	51 (44.35)	47 (31.76)	41 (61.19)	< 0.001
No	170 (65.64)	64 (55.65)	101 (68.24)	26 (38.81)	

## Discussion

VVC was documented as the highest vaginal infection with prevalence rate of 36.5% in this study. The reported rate among pregnant women compares well with 34.2% in Accra, 36.0% in Southwestern Nigeria, 37.4% in Turkey [27, 31, 32]. However, in other studies conducted among pregnant women, the prevalence found in the present study was slightly higher than 26.0 and 30% in Ibadan and Newi Town of Anambra State both in Nigeria [9, 33]. A lower rate of 21% was recorded in Kumasi among women attending gynecological clinic [34] and 22.7% in pregnant women in Burkina Faso [35]. The lower rate of 21% reported by Abruquah et al. [34], study might be due to the small sample size of participants compared with the present study.

In another related study among pregnant women in the United Kingdom, *Candida spp* prevalence was 12.5% as compared to 36.5% in this study [36]. This disparity in the prevalence might be due lack of proper sanitary conditions in many rural communities such as Kintampo compared to the United Kingdom. Risk factors for the infection and local population dynamics accounts for the huge disparities in the prevalence rate across countries.

The high *Candida spp* colonization/infection of the study participants’ vagina could have been due to their pregnancy status. Although, about 20 to 50% of women habour candida species without showing symptoms [7], pregnancy plays a major role in colonization and infection. Leli et al., study demonstrates frequent colonization of the vagina of pregnant women with *Candida spp* compared to non-pregnant women [37]. This is as a result of the high concentration of estrogen during pregnancy which provides a favourable environment for the growth of *Candida spp* [38]. Nonetheless, this high rate calls for urgent attention to the infection since studies suggest possible association between *Candida spp* colonization and higher rate of preterm and low birth weight [37, 39]. Screening for VVC during pregnancy may reduce the risk of preterm delivery [40, 41]. Moreover, symptomatic cases of VVC could cause a lot of discomfort to pregnant women. VVC could also be an indication of an underlying infection such as diabetes mellitus.

The prevalence of BV (30.9%) in pregnant women reported in this study was higher compared with that (1.4%) reported by Apea-Kubi et al., in 2006 among women attending antenatal and gynaecological clinic in Accra [27]. However, it was comparable to a prevalence of 28.0% reported among non-pregnant women in the coastal area of Ghana [42]. Lassey et al., in 2004 reported a prevalence of 47.0% among women with incomplete abortion in Accra [43]. This high rate was probably due to the complication of the abortion which could alter the normal flora of the vagina. Pepin et al., 2011 reported an aggregate rate of 54% on women presenting with vaginal discharge in five West African states including Ghana [18]. This high rate is due to the study’s selection of women with vaginal discharge which is usually a symptom of vaginal infection. In addition, the study used molecular method which is highly sensitive compared to the Nugent criteria for the diagnoses of BV.

In other West African states, the prevalence of BV of 64.3, 17.3 and 6.4% were reported in Southwestern-Nigeria [44], Northeastern-Nigeria [45] and Burkina Faso [19] respectively in pregnant women. Geographical distribution, vaginal hygiene practices and systematic difference in the various populations sampled could account for the variation in the prevalence values. Douching which has been known to be a major risk factor to BV in several cross-sectional and longitudinal studies [46–48] was not associated with BV in this study. This finding is consistent with Demba et al., 2005 and Bukusi et al., 2006 studies which showed no significant relationship between BV and douching [49, 50]. Douching alters the pH of the vagina due to the depletion of lactic acid producing lactobacillus depending on the type of douching solution used.

Prevalence of Trichomonas infection was the lowest (1.4%) compared to the other vaginal infections. Generally, the prevalence of TV found in this study is lower than other studies conducted in Ghana among pregnant women. Apea-Kubi et al., and Adu-Sakordie et al., reported prevalence rates of 2.7% [27] and 5.4% [26] respectively among pregnant women. In the study by Adu-Sakordie et al., a more sensitive latex agglutination kit was used compared to wet mount used in this study. Low rates of TV were recorded in Nigeria (0.5%), Burkina (1.5%) among pregnant women which is of similar finding to this study [17, 51]. Some other studies in women visiting gynaecological and STI clinics recorded rates of 13.2 and 18.1% by wet mount method and polymerase chain reaction (PCR) respectively for TV [28]. This high rate is due to the selection of women visiting STI clinic for treatment of symptoms of vaginal infection.

The low prevalence of TV in the present study may be attributed to the test method used for this study. A study by Richard Asmah et al., concluded wet mount method had very low sensitivity in detecting TV using (PCR) as the gold standard [28, 52]. Collins Adjei et al., study among symptomatic participants recorded prevalence of 1.7, 5.0 and 7.2% with wet mount, culture and enzyme-linked immunosorbent assay (ELISA) methods respectively [53]. These studies confirm the low sensitivity of the wet mount method which could have accounted for the low prevalence of TV in this study. The time between samples collection and analysis could affect the likelihood of detecting TV. In this study, the time between sample collection and analysis was within the recommended 15 min, ruling out the possibility of time playing a role in the low prevalence of TV.

Sexual behavior of the participants which is the main determinant of trichomoniasis may be a factor for the low rate. This is evident in the fact that only one out of the 593 participants interviewed reported having more than one sexual partner in the past 3 months (Table 1). In addition, majority of the participants were married and may not be likely to have multiple sexual partner compared to studies in sex workers.

The study assessed the risk factors for the vaginal infections. After adjusting for other confounding variable, having previous pregnancies of 5 or more (>gravidae 5) (*p* = 0.001) lowered the risk of BV. The protection observed among multigravida women (5 or more) against BV could be due to health education provided during antenatal visits and personal experiences of these 131 pregnant women (Table 1) in previous pregnancies.

Pregnant women in the third trimester were 35 and 53% less likely to have BV (Table 3) compared with the second and first trimesters respectively. BV appeared to decrease with increasing gestational age. This finding is consistent with Water et al., 2008 study which reported BV status decreases as pregnancy progresses [54]. Studies have shown reduced sexual desire and sexual activity as pregnancy ages [55, 56], which indicates reduced sexual frequency. Frequency of sexual intercourse is attributed to be a critical factor to having BV [57] which might account for the decreased BV prevalence with gestational age.

Risk factors considered for VVC and TV in this study were not significantly associated with VVC or TV (*P* > 0.05). Studies have shown recent antibiotic intake and douching to have a positive correlation to VVC [58]. This happens when antibiotic/douching substance kills or suppresses the *Lactobacillus species* which serves as a protective organism making way for the yeast to thrive and colonise the vagina. This negative correlation between antibiotic use and VVC in the present study could be because of the low level of antibiotic intake (4.4%) by the study participants as shown in Table 1.

Asymptomatic participants (Table 4) comprised of more than half of those who tested for BV only (55.7%) and VVC only (68.2%). This highlights the inadequacy of using syndromic management in treating patients. It stresses the importance of testing to know the aetiologic agent as opposed to managing using syndromic approach. Testing to know the aetiologic agent is hindered in many less resourced countries due to lack of adequate laboratory infrastructure.

There have been issues of the need to treat or not to treat asymptomatic vaginal infection in pregnant women arising out of conflicting findings of the benefits or otherwise of the treatment. Many studies have link BV and *Candida spp* colonization to adverse pregnancy outcomes [5, 6]. Unraveling this mystery requires further large scale longitudinal and follow up studies to establish the effect of vaginal infections in pregnancy.

The study’s limitation was the use wet mount method to detection of TV for the study compared with more sensitive method such as PCR. The wet mount method used played a role in the low prevalence of TV. However, some studies had high prevalence for TV using the wet mount method [28] which might be due to the population of women for which the study was carried out on.

## Conclusion and recommendations

The study area had very high prevalence of vaginal infections among pregnant women especially VVC and BV. Having more than four (4) previous pregnancies and in the third trimester of pregnancy lowered the risk for having BV.

Considering the high prevalence of vaginal infections, we recommend that pregnant women attending antenatal clinic should have prompt and adequate laboratory investigations with appropriate treatment to prevent possible adverse effect of the infection on mother and/or foetus.

The uncertainty surrounding how the infection affects the mother and foetus need to be investigated. Therefore, further longitudinal and follow-up studies to investigate the effects of vaginal infections on pregnancy outcomes are recommended.

## Data Availability

Datasets generated during the current study and for this manuscript are available from the corresponding author on reasonable request.

## Abbreviations

ANC: Antenatal care
BV: Bacterial vaginosis
ELISA: Enzyme-linked immunosorbent assay
GCP: Good Clinical Practice
HIV: Human Immune-deficiency Virus
HVS-2: Herpes simplex virus type-2
KHRC: Kintampo Health Research Centre
KHRC-IEC: Kintampo Health Research Centre Institutional Ethics Committee
KMH: Kintampo Municipal Hospital
PCR: Polymerase chain reaction
PID: Pelvic inflammatory disease
PROM: Premature rupture of membrane
ReTI: Reproductive tract infection
TV: Trichomoniasis
VVC: Vulvovaginal candidiasis
WHO: World Health Organization

## References

[1] WHO (2005). Sexually transmitted and other reproductive tract infections: a guide to essential practice.

[2] Marrazzo JM (2011). Interpreting the epidemiology and natural history of bacterial vaginosis: are we still confused?. Anaerobe.

[3] Hillier SL (1995). Association between bacterial vaginosis and preterm delivery of a low-birth-weight infant. The vaginal infections and prematurity study group. N Engl J Med.

[4] Klebanoff MA (2005). Is bacterial vaginosis a stronger risk factor for preterm birth when it is diagnosed earlier in gestation?. Am J Obstet Gynecol.

[5] Leitich H (2003). Bacterial vaginosis as a risk factor for preterm delivery: a meta-analysis. Am J Obstet Gynecol.

[6] Leitich H, Kiss H (2007). Asymptomatic bacterial vaginosis and intermediate flora as risk factors for adverse pregnancy outcome. Best Pract Res Clin Obstet Gynaecol.

[7] Sobel JD (2007). Vulvovaginal candidosis. Lancet.

[8] McClelland RS (2009). Prospective study of vaginal bacterial flora and other risk factors for vulvovaginal candidiasis. J Infect Dis.

[9] Donbraye-Emmanuel O (2010). Detection and prevalence of Candida among pregnant women in Ibadan, Nigeria. World Appl Sci J.

[10] Alli J (2011). Detection and prevalence of Candida isolates among patients in Ibadan, Southwestern Nigeria. J Microbiol Biotech Res.

[11] Hillier SL (1993). Diagnostic microbiology of bacterial vaginosis. Am J Obstet Gynecol.

[12] Sobel JD (2005). What’s new in bacterial vaginosis and trichomoniasis?. Infect Dis Clin N Am.

[13] Redelinghuys MJ (2016). Normal flora and bacterial vaginosis in pregnancy: an overview. Crit Rev Microbiol.

[14] Sobel JD (2000). Bacterial vaginosis. Annu Rev Med.

[15] Cherpes TL (2003). Association between acquisition of herpes simplex virus type 2 in women and bacterial vaginosis. Clin Infect Dis.

[16] Donders G (2010). Diagnosis and management of bacterial vaginosis and other types of abnormal vaginal bacterial flora: a review. Obstet Gynecol Surv.

[17] Kirakoya-Samadoulougou F (2008). Bacterial vaginosis among pregnant women in Burkina Faso. Sex Transm Dis.

[18] Pepin J (2011). The complex vaginal flora of West African women with bacterial vaginosis. PLoS One.

[19] Jespers V (2014). Prevalence and correlates of bacterial vaginosis in different sub-populations of women in sub-Saharan Africa: a cross-sectional study. PLoS One.

[20] Kirakoya-Samadoulougou F (2011). Epidemiology of herpes simplex virus type 2 infection in rural and urban Burkina Faso. Sex Transm Dis.

[21] Martin HL (1999). Vaginal lactobacilli, microbial flora, and risk of human immunodeficiency virus type 1 and sexually transmitted disease acquisition. J Infect Dis.

[22] Atashili J (2008). Bacterial vaginosis and HIV acquisition: a meta-analysis of published studies. AIDS.

[23] Organization, W.H. and W.H. Organization (1995). An overview of selected curable sexually transmitted diseases. Global program on AIDS.

[24] Workowski KA (2010). Sexually transmitted diseases treatment guidelines, 2010. MMWR Recomm Rep.

[25] Sena AC (2007). Trichomonas vaginalis infection in male sexual partners: implications for diagnosis, treatment, and prevention. Clin Infect Dis.

[26] Adu-Sarkodie Y (2004). Comparison of latex agglutination, wet preparation, and culture for the detection of trichomonas vaginalis. Sex Transm Infect.

[27] Apea-Kubi KA (2006). Bacterial vaginosis, Candida albicans and trichomonas vaginalis infection in antenatal and gynaecological patients in Ghana. Trop J Obstet Gynaecol.

[28] Squire DS (2019). Trichomonas vaginalis infection in southern Ghana: clinical signs associated with the infection. Trans R Soc Trop Med Hyg.

[29] Nugent RP, Krohn MA, Hillier SL (1991). Reliability of diagnosing bacterial vaginosis is improved by a standardized method of gram stain interpretation. J Clin Microbiol.

[30] Apea-Kubi KA (2005). Bacterial vaginosis, Candida albicans and trichomonas vaginalis infection in antenatal and gynaecological patients in Ghana. Trop J Obstet Gynaecol.

[31] Olowe O (2014). Prevalence of vulvovaginal candidiasis, trichomoniasis and bacterial vaginosis among pregnant women receiving antenatal care in Southwestern Nigeria. Eur J Microbiol Immunol.

[32] Guzel AB (2011). An evaluation of risk factors in pregnant women with Candida vaginitis and the diagnostic value of simultaneous vaginal and rectal sampling. Mycopathologia.

[33] Okonkwo N, Umeanaeto P. Prevalence of vaginal candidiasis among pregnant women in Nnewi Town of Anambra State, Nigeria. Afr Res Rev. 2010;4(4).

[34] Abruquah H (2012). Prevalence and antifungal susceptibility of Candida species isolated from women attending a gynaecological clinic in Kumasi, Ghana. J Sci Technol (Ghana).

[35] Sangaré I., Sirima C., Bamba S., Zida A., Cissé M., Bazié W.W., Sanou S., Dao B., Menan H., Guiguemdé R.T. (2018). Prevalence of vulvovaginal candidiasis in pregnancy at three health centers in Burkina Faso. Journal de Mycologie Médicale.

[36] Akinbiyi A, Watson R, Feyi-Waboso P (2008). Prevalence of Candida albicans and bacterial vaginosis in asymptomatic pregnant women in South Yorkshire, United Kingdom. Arch Gynecol Obstet.

[37] Leli C (2013). Association of pregnancy and Candida vaginal colonization in women with or without symptoms of vulvovaginitis. Minerva Ginecol.

[38] Garcia Heredia M (2006). Prevalence of vaginal candidiasis in pregnant women. Identification of yeasts and susceptibility to antifungal agents. Rev Argent Microbiol.

[39] Holzer I (2017). The colonization with Candida species is more harmful in the second trimester of pregnancy. Arch Gynecol Obstet.

[40] Kiss H, Petricevic L, Husslein P (2004). Prospective randomised controlled trial of an infection screening programme to reduce the rate of preterm delivery. BMJ.

[41] Roberts CL (2011). Treatment of asymptomatic vaginal candidiasis in pregnancy to prevent preterm birth: an open-label pilot randomized controlled trial. BMC Pregnancy Childbirth.

[42] Aubyn GB, Tagoe DNA (2013). Prevalence of vaginal infections and associated lifestyles of students in the university of Cape Coast, Ghana. Asian Pac J Trop Dis.

[43] Lassey A (2004). Potential pathogens in the lower genital tract at manual vacuum aspiration for incomplete abortion in Korle Bu Teaching Hospital, Ghana. East Afr Med J.

[44] Ajani G (2012). Nugent scores of pregnant women in a tertiary institution in Nigeria.

[45] Ibrahim S (2014). Prevalence of bacterial vaginosis in pregnant women in Maiduguri, North-Eastern Nigeria. Niger J Clin Pract.

[46] Trabert B, Misra DP (2007). Risk factors for bacterial vaginosis during pregnancy among African American women. Am J Obstet Gynecol.

[47] Luong M-L (2010). Vaginal douching, bacterial vaginosis, and spontaneous preterm birth. J Obstet Gynaecol Can.

[48] Durugbo II (2015). Bacterial vaginosis among women with tubal factor infertility in Nigeria. Int J Gynecol Obstet.

[49] Bukusi EA (2006). Bacterial vaginosis: risk factors among Kenyan women and their male partners. Sex Transm Dis.

[50] Demba E (2005). Bacterial vaginosis, vaginal flora patterns and vaginal hygiene practices in patients presenting with vaginal discharge syndrome in the Gambia, West Africa. BMC Infect Dis.

[51] Sunday-Adeoye I (2009). The prevalence of trichomonas vaginalis and Candida albicans infection in the lower genital tracts of antenatal patients in Abakaliki, Southeastern Nigeria. Nepal J Obstet Gynaecol.

[52] Asmah RH (2018). Trichomonas vaginalis infection and the diagnostic significance of detection tests among Ghanaian outpatients. BMC Womens Health.

[53] Adjei C (2019). Prevalence and the evaluation of culture, wet mount, and ELISA methods for the diagnosis of trichomonas vaginalis infection among Ghanaian women using urine and vaginal specimens. Trop Med Health.

[54] Waters TP (2008). Longitudinal trajectory of bacterial vaginosis during pregnancy. Am J Obstet Gynecol.

[55] Bartellas E (2000). Sexuality and sexual activity in pregnancy. BJOG Int J Obstet Gynaecol.

[56] Uwapusitanon W, Choobun T (2004). Sexuality and sexual activity in pregnancy. J Med Assoc Thail.

[57] Verstraelen H (2010). The epidemiology of bacterial vaginosis in relation to sexual behaviour. BMC Infect Dis.

[58] Ahmad A, Khan AU (2009). Prevalence of Candida species and potential risk factors for vulvovaginal candidiasis in Aligarh, India. Eur J Obstet Gynecol Reprod Biol.

